# Deconvolution of octahedral Pt_3_Ni nanoparticle growth pathway from in situ characterizations

**DOI:** 10.1038/s41467-018-06900-z

**Published:** 2018-10-26

**Authors:** Xiaochen Shen, Changlin Zhang, Shuyi Zhang, Sheng Dai, Guanghui Zhang, Mingyuan Ge, Yanbo Pan, Stephen M. Sharkey, George W. Graham, Adrian Hunt, Iradwikanari Waluyo, Jeffrey T. Miller, Xiaoqing Pan, Zhenmeng Peng

**Affiliations:** 1 Department of Chemical and Biomolecular Engineering, The University of Akron, Akron, OH 44325 USA; 2 Department of Materials Science and Engineering, University of Michigan, Ann Arbor, MI 48109 USA; 3 Department of Materials Science and Engineering, University of California-Irvine, Irvine, CA 92697 USA; 4 Davidson School of Chemical Engineering, Purdue University, West Lafayette, IN 47907 USA; 5 National Synchrotron Light Source II, Brookhaven National Laboratory, Upton, NY 11973 USA; 6 Department of Physics and Astronomy, University of California-Irvine, Irvine, CA 92697 USA

## Abstract

Understanding the growth pathway of faceted alloy nanoparticles at the atomic level is crucial to morphology control and property tuning. Yet, it remains a challenge due to complexity of the growth process and technical limits of modern characterization tools. We report a combinational use of multiple cutting-edge in situ techniques to study the growth process of octahedral Pt_3_Ni nanoparticles, which reveal the particle growth and facet formation mechanisms. Our studies confirm the formation of octahedral Pt_3_Ni initiates from Pt nuclei generation, which is followed by continuous Pt reduction that simultaneously catalyzes Ni reduction, resulting in mixed alloy formation with moderate elemental segregation. Carbon monoxide molecules serve as a facet formation modulator and induce Ni segregation to the surface, which inhibits the (111) facet growth and causes the particle shape to evolve from a spherical cluster to an octahedron as the (001) facet continues to grow.

## Abstract (web summary)

Understanding the growth pathway of faceted alloy nanoparticles at the atomic level is crucial to morphology control and property tuning, but remains a challenge. Here, the authors reveal the particle growth and facet formation mechanisms of octahedral Pt_3_Ni nanoparticles using multiple cutting-edge in situ techniques.

## Introduction

Faceted platinum alloy nanoparticles, in particular octahedral Pt–Ni, have been recognized as one of the most promising cathode catalysts for use in hydrogen fuel cells for their dramatically improved oxygen reduction reaction (ORR) activity compared to their spherical counterparts and pure Pt^1–4^. These findings revealed significant catalytic structure-property correlations and suggested the importance of controlling the facet growth of nanoparticles to achieve the desired property. Although considerable success has been achieved in synthesizing faceted alloy nanoparticles after years of intensive research, these achievements were mostly empirical. Knowledge of the growth pathway, which is essential to guide rational synthesis, is still lacking. The past decade has witnessed technological advances of in situ electron microscopy and its use in obtaining the growth trajectory of pure metal nanoparticles^5–12^. Compared with pure metals, the growth pathway of faceted alloy nanoparticles is more complicated. With multiple elements being involved, it is challenging to identify their behaviors, both individually as different elements and collectively as alloy components, during the particle growth^13,14^.

Herein we report a combinational use of several cutting-edge in situ characterization techniques, including aberration-corrected scanning transmission electron microscopy (AC-STEM), ambient pressure X-ray photoelectron spectroscopy (AP-XPS), and X-ray absorption spectroscopy (XAS), to study the growth process of octahedral Pt_3_Ni nanoparticles. By integrating the information obtained from these techniques, together with computational simulations, a clear pathway for the particle growth and facet formation is revealed.

## Results

### In situ STEM characterizations

**Fig. 1:**
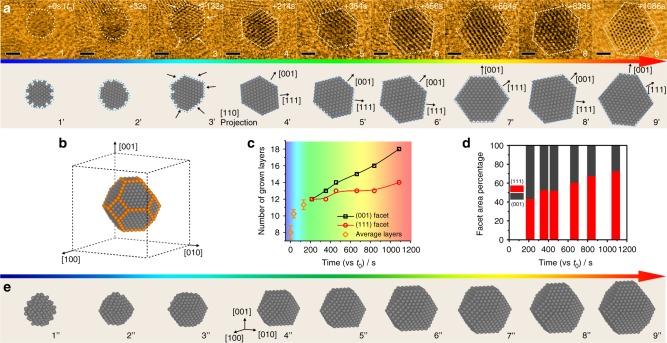
Shape evolution of the growing Pt_3_Ni nanoparticle. **a** In situ STEM images of one growing Pt_3_Ni nanoparticle (upper row) and corresponding 2D projection model (lower row), scale bar: 1 nm. **b** Representative atomic model (not to scale) of the nanoparticle during growth. **c** Number of grown layers of the (001) and (111) facets (Error bars correspond to standard deviations of at least three independent layer estimation) and **d** their surface exposure ratio as function of experimental time. **e** 3D model (not to scale) of the Pt_3_Ni growth process. The early stage model 1” is only constructed to show a possible dynamical cluster structure

Direct visualization of the growth process of octahedral Pt_3_Ni was performed on a JEOL-JEM 3100R05 microscope that is equipped with a Protochips Atmosphere^TM^ gas cell system (Supplementary Fig. 1)^15,16^, realizing dynamic observation at the atomic scale under atmospheric pressures. The reaction condition for growing octahedral Pt_3_Ni followed a solid-state chemistry method we recently developed, in which H_2_ acts as a reductant and CO acts as a capping agent for morphology control^1^. Figure 1a shows the time-sequenced STEM images of one single particle at its different growth stages (top row). Simulated images, presented in the bottom row, were obtained based on the experimental data. It was quite challenging to image the particles clearly at their early growth stage (denoted as *t*_0_ = 0 s), primarily because of their high structural instability and mobility in the small cluster size range. Moreover, the use of low-dose STEM imaging condition in this study, which was necessary for minimizing the electron beam effect, sacrificed the image quality to some extent (Supplementary Note 1, Supplementary Fig. 2). Nevertheless, the observed sphere-like characteristic indicated no apparent facet formation of the particles and highly dynamic property in their early growth stage, which has been discussed in supplementary materials (Supplementary Note 2). Facet formation and distinct lattice fringes were observed to evolve when the particle size slightly exceeded 1 nm. Two groups of facets, i.e., {111} and {001}, were indexed as indicated by the atomic model in Fig. 1b. Noticeably, the {111} facets exhibited a significantly slower growth rate compared with the {001} under the reaction condition, which was evidenced by a smaller number of atomic layers along the [111] axis than along the [001] (Fig. 1c). Our previous study has identified the important role of CO molecules in inhibiting the Pt_3_Ni {111} growth^1^, which led to the formation of octahedral morphology rather than a spheroid one. The slower {111} growth resulted in the gradual domination of this group of facet planes and diminishment of the {001} over the reaction time (Fig. 1d, Supplementary Fig. 3), which was accompanied by a continuous morphological change of the growing particle. The in situ observation of other growing particles found a same trend (Supplementary Figs. 4–6) with a homogenous distribution of the particles during the growth (Supplementary Fig. 7) and a high yield of octahedral morphology in the final product (Supplementary Fig. 8), suggesting this is a generic pathway for octahedral Pt_3_Ni growth (a more detailed discussion can be found in the Supplementary Note 2, Supplementary Fig. 9). Thus we constructed three-dimensional (3D) models based on the in situ STEM data to better illustrate the growth process of octahedral Pt_3_Ni (Fig. 1e), when there is a continual increase in size along with a gradual morphological transition from spherical cluster to polyhedron and finally to octahedron.

### In situ AP-XPS measurements

**Fig. 2:**
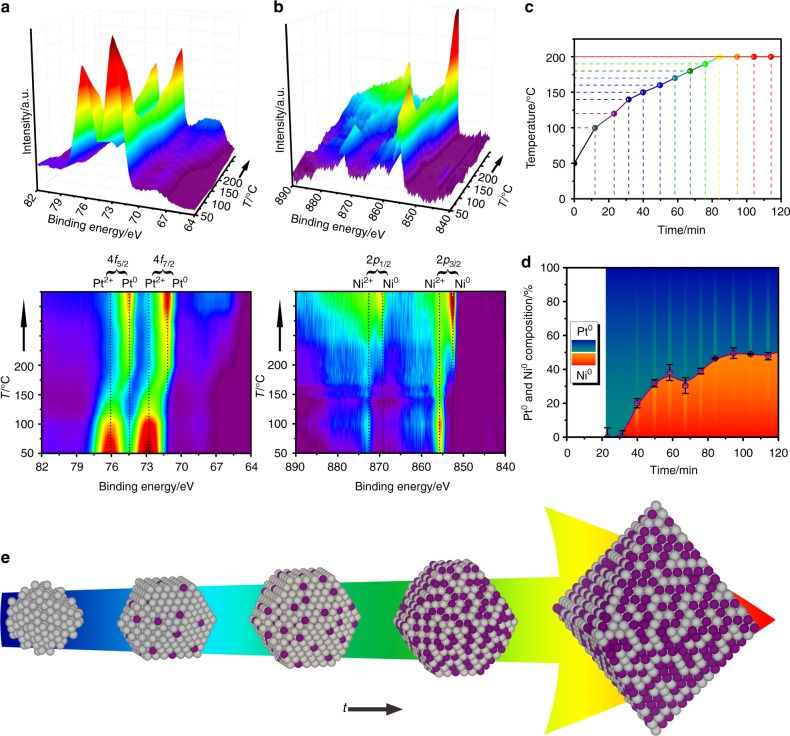
Surface composition evolution of the growing Pt_3_Ni nanoparticles. **a** Pt 4 *f* and **b** Ni 2*p* AP-XPS spectra and corresponding 2D projections of growing particles. **c** Temperature profile as a function of experiment time. **d** Calculated Pt^0^ and Ni^0^ contents near surface region of the growing particles based on quantitative XPS analysis (Error bars correspond to standard deviations of at least three independent composition analysis). **e** Growth model (not to scale) of Pt_3_Ni nanoparticle with near surface Pt^0^/Ni^0^ ratio changes (gray: Pt; purple: Ni)

In situ AP-XPS experiments were conducted to characterize the elemental distribution near the particle surface region through the growth process by continuously collecting Pt 4*f* and Ni 2*p* signals under the synthesis condition (see experimental details in Methods section, Supplementary Fig. 10). Figure 2a, b shows the original Pt 4*f* and Ni 2*p* spectra collected according to the temperature profile in Fig. 2c. Peaks at 72.8 and 76.1 eV, corresponding to Pt^2+^ 4*f*_7/2_ and 4*f*_5/2_^17^, and peaks at 855.7 and 873.1 eV, corresponding to Ni^2+^ 2*p*_3/2_ and 2*p*_1/2_^18^, were identified prior to the experiments, indicating that both elements remained in their precursor state. A new peak emerged at 71.7 eV when the temperature was raised to around 120 °C. This new peak was assigned to Pt^0^ 4*f*_7/2_, with its emergence suggesting an initiation of Pt reduction. Gradual growth of the Pt^0^ 4*f*_7/2_ peak and diminishment of the Pt^2+^ 4*f*_7/2_ peak were observed with a further increase in the temperature, which indicated a continual Pt reduction process. Ni exhibited a delayed reduction compared to Pt, because the Ni^0^ 2*p*_3/2_ peak at 852.7 eV did not appear until the temperature was above 150 °C. Control experiments were conducted with pure Pt(acac)_2_ and Ni(acac)_2_ to characterize their individual reduction processes (Supplementary Figs. 11, 12). The onset reduction temperature for Pt(acac)_2_ remained at around 120 °C, whereas Ni(acac)_2_ cannot be effectively reduced until above 200 °C. This interesting observation suggested that reduced Pt served as a catalyst for Ni reduction.

Quantitative analysis of the AP-XPS data set (Supplementary Notes 3–5) yielded more detailed composition information of the growing particles in their near surface region (Fig. 2d), which provided an important basis for understanding the particle growth pathway. Because Pt reduction initiated at around 120 °C and no metallic Ni was detected until 150 °C, it could be concluded that Pt, rather than a Pt–Ni alloy, constituted the particles at their nucleation and early growth stage and served as seeds for the octahedral Pt_3_Ni formation. Above 150 °C, Ni began to be effectively reduced, as evidenced by a rapid increase in the Ni^0^ content. In addition, a gradual shift of Pt^0^ 4*f*_7/2_ peak from 71.7 to 71.2 eV was observed in this stage (Fig. 2a), which can be attributed to electron transfer from Ni^0^ to Pt^0^ and was consistent with the quantitative surface composition change^19^. Interestingly, the near surface Ni^0^ content gradually reached nearly 50 at% at the late particle growth stage, which was significantly higher than its average content of 25 at% in the final Pt_3_Ni product. This discrepancy suggested the particles were Ni-enriched in the surface region, which was in good agreement with previous studies in literature^20^. Another interesting observation was that the surface Ni content, despite of its higher onset reduction temperature than Pt, reached 25 at% soon after its reduction and remained above this value until completion of the reduction process. It revealed, besides Ni reduction, that Ni in the bulk likely underwent a continual segregation to the surface region during the particle growth process. By combining the information obtained from STEM and AP-XPS, we were able to depict both morphological and surface composition changes of the growing particles. As illustrated in Fig. 2e, the growth of octahedral Pt_3_Ni starts with the formation of Pt nuclei and small spherical clusters, which continues to evolve through polyhedron to octahedron in morphology and evolve from pure Pt to about Pt_50_Ni_50_ in surface composition.

### In situ XAS analysis

**Fig. 3:**
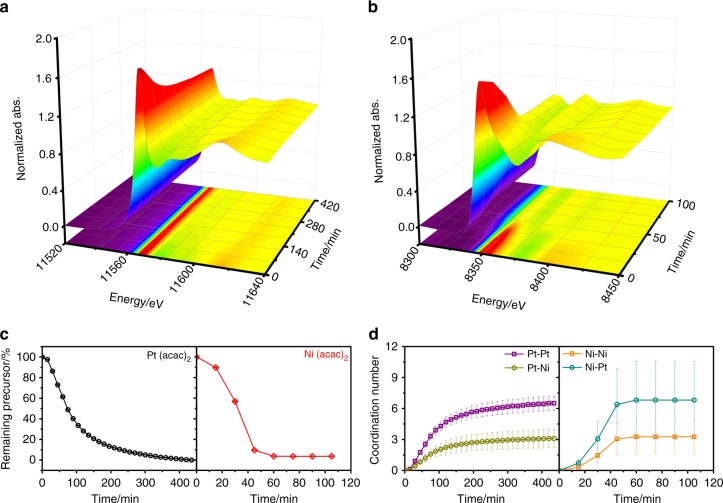
Overall composition changes of the growing particles. **a** Pt L_3_-edge and **b** Ni K-edge XAS spectra of the growing nanoparticles. **c** Percentage of remaining precursors calculated from the XAS data and **d** coordination numbers of the reduced elements calculated from the XAS data as function of reaction time (Error bars correspond to fitting errors of XAS data processing)

In situ XAS experiments were carried out to trace overall composition changes of the growing particles (Supplementary Notes 4, 5, Supplementary Fig. 13)^21,22^. Note that the experiments were conducted at a relatively lower temperature of 160 °C compared to that in AC-STEM and AP-XPS experiments so that the particle growth kinetics was sufficiently slow to allow for in situ XAS measurements, given that it took approximately 15 min to collect one XAS spectrum (see Methods section for experimental details). Our control experiments confirmed that such a change in reaction temperature altered the particle growth kinetics but would not affect the final particle morphology and composition (Supplementary Figs. 14, 15), which validated a combinational use of the in situ XAS data. Pt L_3_-edge and Ni K-edge spectra were collected in time series during the particle growth process (Fig. 3a, b and Supplementary Fig. 15). The extents of Pt and Ni reduction were evaluated based on changes in their XANES spectra. Figure 3c shows the plot of the percentage of remaining precursors at different reaction time. It was apparent that Ni reduction occurred more rapidly than Pt once it was initiated, as shown by a shorter period of time before the precursor depletion. The control XAS experiments with Ni(acac)_2_ showed little Ni reduction by itself under the same experimental condition (Supplementary Fig. 16). The results were in good agreement with the AP-XPS findings, which further supported our conclusion that Ni reduction was catalyzed by Pt. The completion of Ni reduction in a shorter time than that for Pt could lead to an argument that the grown particles should have Pt-enriched surfaces, which contradicted the AP-XPS observation of Ni enrichment in the surfaces. This contradiction can be resolved on the basis of Ni segregation to the surface during the octahedral Pt_3_Ni growth, as revealed by the AP-XPS data. This explanation was supported by post-treatment experiments with octahedral PtNi alloys in previous studies, which reported significant surface Ni segregation upon exposure to CO atmosphere at elevated temperatures^23^. Average coordination numbers for reduced Pt and Ni during the particle growth were calculated from the XAS data (Fig. 3d, Supplementary Table 1, Supplementary Fig. 17). The Pt–Pt coordination number remained higher than that of Pt–Ni throughout the reaction, and both gradually increasing with time. In comparison, the Ni-Pt coordination number was higher than that of Ni-Ni coordination and they seemed stabilized after the completion of the Ni reduction. The values of both ratios (Pt–Pt/Pt–Ni and Ni–Pt/Ni–Ni) in the octahedral Pt_3_Ni deviated from the Pt/Ni atomic ratio (i.e., 3:1) to a moderate extent. Considering the Pt enrichment in core region of the particles also contributed to such deviations, the results indicated that although Ni atoms were enriched on the surface, they were relatively well mixed with Pt atoms in the lattice.

### First principle calculation of CO preferential adsorption

**Fig. 4:**
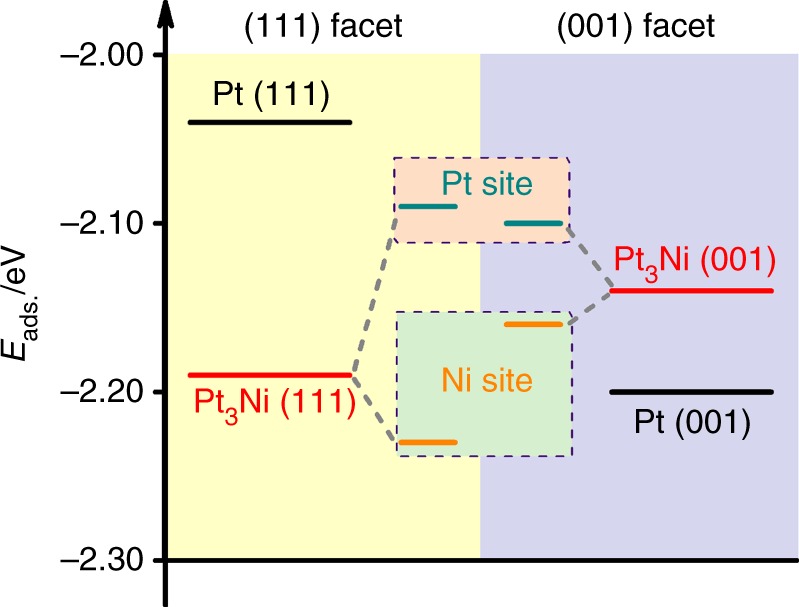
CO adsorption energy on different facets. DFT calculation of CO adsorption energy on the (111) and (001) facets of surface Ni-enriched Pt_3_Ni

To understand the facet formation mechanism and the possible influences of Ni surface segregation on the facet formation, density functional theory (DFT) simulations were performed to substantiate our conjecture (Supplementary Fig. 18). This approach has already been used to effectively explain the morphology control mechanism under synthesis conditions^5,24,25^. The results showed that the enrichment of surface Ni significantly enhanced CO adsorption on the Pt_3_Ni (111) plane, which led to the formation of an octahedral particle (Fig. 4 and Supplementary Table 2). Compared with CO adsorption on Pt (111) (*E*_ads_ = −2.04 eV), the adsorption of CO molecules was greatly strengthened at both Pt (−2.09 eV) and Ni (−2.23 eV) sites on Ni-enriched Pt_3_Ni (111). Although the *E*_ads_ of CO at Pt site (−2.10 eV) on Ni-enriched Pt_3_Ni (001) was comparable with that at Pt site on the (111), the value at Ni site on the (001) facet (−2.16 eV) was less negative than that at the counterpart Ni site. The *E*_ads_ of CO on Ni-enriched Pt_3_Ni (111) was about 0.05 eV more negative than that on the (001) on average. Although the energy difference was not dramatic, it altered the favored adsorption facet from the (001) planes for pure Pt to the (111) planes for Ni-enriched Pt_3_Ni that contributed to an octahedral morphology formation. Noticeably, DFT calculations showed that CO still preferred to adsorb on Pt_3_Ni (001) if there was no Ni enrichment at the surface (Supplementary Fig. 19), which further supported the crucial role of surface Ni enrichment in Pt_3_Ni for octahedral shape formation. Apart from the CO preferential adsorption, previous report indicates that the facet-to-facet atom diffusion would also play a role to shape the morphology owing to the different diffusion barriers^26^. Thus besides the observed dominant role of CO in controlling the octahedral Pt_3_Ni growth, it would be possible that surface atom diffusion on the growing particles also contributes to the facet formation.

## Discussion

**Fig. 5:**
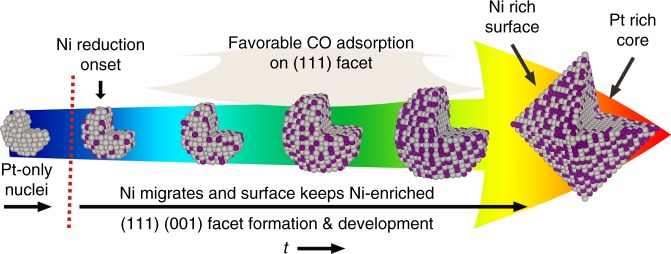
Schematic octahedral Pt_3_Ni growth pathway. Scheme (not to scale) of octahedral Pt_3_Ni growth pathway with inner section view (gray: Pt; purple: Ni). The nuclei stage is only constructed to show a possible dynamical structure

A complete picture of the growth mechanism of octahedral Pt_3_Ni can be drawn by considering all of the information gained from the in situ experiments and DFT simulations. The STEM images with acceptable quality (identifiable lattice fringes and facets), except the early stage cluster, allowed us to build up the structure models. And XAS characterizations provided more detailed information like the atom distribution providing a clear picture of the growth pathway. Specifically, AP-XPS indicated a Pt-only stage, and both AP-XPS and XAS experiments confirmed the migration of Ni and Ni-rich-surface/Pt-rich-core properties. And the DFT calculations revealed the favorable CO adsorption on Ni-enriched Pt_3_Ni (111) facet. As shown in Fig. 5, Pt nuclei (early stage clusters) are first generated under the synthesis condition, which can be attributed to the facile reducibility of the Pt precursor. Ni then becomes catalytically reduced by Pt, leading to a mixed alloy formation. The continued reduction of Pt and Ni results in the growth in particle size and the evolution of low-index (111) and (001) facets. Along with the particle growth, Ni segregates outwards induced by CO and enriches the surface. CO molecules adsorb preferentially on the Ni-enriched (111) surfaces, which decreases their growth rate. This leads to the gradual growth of the (111) surfaces and diminishment of the (001) facets, and eventually to the formation of an octahedral morphology.

In conclusion, multiple cutting-edge in situ characterization techniques reveal the growth and facet formation pathway of octahedral Pt_3_Ni nanoparticles. Results indicate the surface enriched Ni plays a significant role in controlling the octahedra development by mediating the CO adsorption on (111) and (001) facets of Pt_3_Ni nanoparticles. Our findings highlight the significance of advanced in situ techniques in researching catalyst preparation and morphology control, providing an insightful understanding in synthesizing shaped catalysts.

## Methods

### Materials

Platinum acetylacetonate (Pt(acac)_2_, 97%) and nickel acetylacetonate (Ni(acac)_2_, 95%) were purchased from Sigma-Aldrich. Carbon black (C, Vulcan® XC-72R) was purchased from Cabot. Chloroform (CHCl_3_, 99.9%) was purchased from Fisher Scientific.

### Sample preparation

The carbon supported Pt(acac)_2_ and Ni(acac)_2_ sample for in situ experiments was prepared via a dry impregnation method. In a typical procedure, 40 mg Pt(acac)_2_ and 17 mg Ni(acac)_2_ were dissolved in 3 mL chloroform, then the solution was added drop wisely onto 80 mg pretreated XC-72 carbon (300^o^C, overnight) under vigorous stirring. After the impregnation, the mixed sample (20 wt.% Pt loading, Pt:Ni = 3:1, molar ratio) was sealed in a glass vial and ready for the in situ characterizations. For comparison, the carbon supported pure Pt(acac)_2_ and pure Ni(acac)_2_ samples were also prepared through the same procedure.

### In situ STEM

In situ scanning transmission electron microscopy (STEM) study of Pt_3_Ni octahedra growth was performed on a double Cs-corrected JEOL-JEM 3100-R05 microscope equipped with the Protochips Atmosphere^TM^ gas cell system. In a typical method, the pre-mixed carbon supported precursor was dispersed in anhydrous methanol under sonication. Then the suspension was dropped directly onto a thermal E-chip and dried under the infrared lamp. The thermal E-chip is equipped with a thin ceramic heating membrane that can be controlled by the Protochips Atmosphere^TM^ system. After that, a second E-chip window was then placed on top of the thermal chip in the holder, and this could create a thin gas chamber sealed from the high vacuum of the TEM column. The sample was situated between two 30–50 nm thick Si_3_N_4_ windows of the E-chips with a 5μm gap in between. The cross-section scheme of the assembled gas cell is shown in Supplementary Fig. 1. The operation accelerating voltage was 300 kV with a small probe current of 20pA, and the heating temperature for the gas cell was 200 °C (based on the Protochips calibration) with high purity H_2_/CO mixture gas (H_2_: 4%, CO: 96%, purity: 99.9995 %) flow at one atmosphere (760 Torr).

### In situ AP-XPS

In situ AP-XPS experiments were performed at the 23-ID-2 (IOS) beamline of the National Synchrotron Light Source II (NSLS-II) at Brookhaven National Laboratory. The end-station was equipped with a differentially pumped hemispherical analyzer (Specs Phoibos 150 NAP), which was offset by 70° from the incident synchrotron light and 20° from the surface normal of the sample (Supplementary Fig. 10). A more detailed description of the beamline and endstation can be found elsewhere^27^. A photon energy of 1100 eV was used for all experiments. The powder sample was pressed onto a tantalum foil, which was clipped onto a ceramic button heater. The temperature was monitored through a type K thermocouple placed underneath the foil. 100mTorr H_2_ and 500mTorr CO (Matheson, ultra-high purity) were introduced into the measurement chamber using variable leak valves, and the pressure was monitored using a capacitance manometer. The collected XPS spectra were calibrated based on the C1*s* = 284.5 eV as a reference. The Pt 4*f* and Ni 2*p* peaks were analyzed using a Shirley-type background with XPSpeak 4.1 software.

### In situ XAS

X-ray absorption measurements were acquired on the bending magnet beamline of the Materials Research Collaborative Access Team (MRCAT) at the Advanced Photon Source, Argonne National Laboratory. Photon energies were selected using a water-cooled, double-crystal Si(111) monochromator, which was detuned by approximately 50% to reduce harmonic reflections. Measurements were made in transmission mode, and data points were acquired in three separate regions (energies relative to the elemental Ni K-edge 8333 eV and Pt L_3_-edge 11564 eV): a pre-edge region (−250 to −30 eV, step size = 10 eV, dwell time = 0.25 s), the XANES region (−30 to +30 eV, step size = 0.5 eV, dwell time = 0.25 s), and the EXAFS region (to 13 Å^−1^, step size = 0.07 Å^−1^, dwell time = 1 s). The ionization chambers were optimized for the maximum current with linear response (~1010 photons detected sec^−1^) 95% N_2_ and 5% Ar in the incident ion chamber and 40% N_2_ and 60% Ar in the transmission detector for both Ni edge and Pt edge measurement. A Ni or Pt foil spectrum was acquired simultaneously with each measurement for energy calibration. Samples were treated in a continuous-flow reactor, which consisted of a quartz tube (1-inch OD, 10-inch length) sealed with Kapton windows by two Ultra-Torr fittings (Supplementary Fig. 13). Ball valves were welded to each Ultra-Torr fitting and served as the gas inlet and outlet. An internal K type thermocouple (Omega) was placed against the sample holder to monitor temperature.

Samples (~10 mg) were pressed into a cylindrical sample holder consisting of six wells, forming a self-supporting wafer. The samples were treated at 160^o^C under H_2_/CO gas (2.5% H_2_, 50% CO and 47.5% Ar, ultra-high purity, 1 atm), and the XAS data were collected in situ.

### XAS data processing

Standard procedures for normalization and background subtraction were performed using Demeter 0.9.25 software package. The edge energy of the X-ray absorption near edge structure (XANES) spectrum was determined from the inflection point in the leading edge, i.e., the maximum in the first derivative of the leading edge of the XANES spectrum. The pre-edge energies were also obtained in the first derivative using the zero-crossing point. The coordination parameters were obtained by a least square fit in R-space, k^2^-weighted Fourier transformed data using Artemis^28^.

### DFT calculations

All DFT simulations were performed using the Quantum ESPRESSO package^29^. Structure relaxation and energy calculation were carried out through generalized gradient approximation (GGA) and the Perdew-Burke-Ernzerhof (PBE) functional with projector-augmented wave (PAW) sets from PSlibrary 0.3.1^30^. The van der Waals (vdW) corrections were also included using the method of “Grimme-D2”^31^. The bulk lattice of Pt and intermetallic Pt_3_Ni were optimized prior to the cleavage of the (111) and (001) surface. The slab models were constructed using six-layer *p*(2 × 2) close-packed surface with a vacuum layer of 20 Å for both (111) and (001) surfaces. For the surface Ni enriched Pt_3_Ni model, we manually substituted the targeted Pt atoms in the upper three layers of Pt slab models and left the lower three layers Ni-free while keeping the overall stoichiometric ratio Pt:Ni = 3:1. For all the slab models, the bottom two layers are fixed during the structure relaxation. And for the CO adsorption, an on-top adsorption site was chosen to make the simulation consistent. We applied a plane-wave function cutoff of 50 Ry with a charge density cutoff of 500 Ry to initiate the calculations, and a k-point mesh of 5 × 5 × 1 was used. All the energies were calculated after the structures were fully relaxed. The adsorption energy for CO was calculated using the equation *E*_ads_ = *E*_system_ − *E*_slab_ − *E*_CO_. The weighted *E*_ads_ for Pt and Ni co-exist surface was defined as *E*_ads,w_ = *E*_ads,Pt_ × *θ*_Pt_ + *E*_ads,Ni_ × *θ*_Ni_, where *θ*_Pt_ and *θ*_Ni_ are the surface coverage of Pt and Ni atoms in top layer.

## Electronic supplementary material

Supplementary Information

## Data Availability

The data that support the findings of this study are available from the corresponding author upon reasonable request.
