# Supplementary material for: Deconvolution of octahedral Pt3Ni nanoparticle growth pathway from in situ characterizations
Source: Nat Commun. 2018 Oct 26;9:4485. doi: 10.1038/s41467-018-06900-z (PMC6203767; doi:10.1038/s41467-018-06900-z)
Supplement: Supplementary file 1 — Supplementary Information [file 41467_2018_6900_MOESM1_ESM.pdf]

Supplementary Information for

Deconvolution of octahedral Pt_3Ni nanoparticle growth pathway from *in situ* characterizations

Xiaochen Shen, Changlin Zhang, Shuyi Zhang, Sheng Dai, Guanghui Zhang, Mingyuan Ge, Yanbo Pan, Stephen M. Sharkey, George W. Graham, Adrian Hunt, Iradwikanari Waluyo*, Jeffrey T. Miller*, Xiaoqing Pan*, Zhenmeng Peng*

*Correspondence to: zpeng@uakron.edu, xiaoqinp@uci.edu, jeffrey-t-miller@purdue.edu, iwaluyo@bnl.gov

Table of Contents

Supplementary Notes 1-5
Supplementary Figures 1-19
Supplementary Tables 1-2
Supplementary References 1-18

Supplementary Notes

Supplementary Note 1: Electron beam effect discussion

In order to minimize the possible electron beam effect, both the gun extraction voltage, and the electrostatic gun lens voltage were reduced to generate a very small probe current 7.0 pA for our *in situ* experiment, while the typical probe current for STEM imaging is usually above 30 pA [1,2]. The acquisition time of image collection was limited to less than 16 seconds per frame to minimize the scanning time under high magnifications. Considering the probe size (1.3 Å) and our imaging condition (a pixel dwell time of 15 μs/pixel and a pixel size of 0.04 $Å^2$), the electron dose we used was about 1.6×10^4 e/$Å^2$, which is about 1~2 orders of magnitude lower than typical dose for STEM imaging [3,4]. In addition, it should be noted that the samples were enclosed in a gas cell. The beam intensity is actually reduced by a factor of approximately 3.0 due to scattering from the Si_3N_4 membranes (about 30-50 nm thick) before it interacts with the sample, reducing the possibility of a beam effect even further [5].

In addition, based on our *in situ* observation, the only beam effect took place at the nucleation stage of the Pt_3Ni nanoparticles. As shown in Supplementary Fig. 2, at the observed area under electron beam illumination, the nucleation speed was accelerated since the electron beam is considered to have a slight reducing effect [6,7]. However, the beam effect is not critical in our *in situ* STEM experiment since the Pt_3Ni nanoparticles were still able to nucleate without the electron beam illumination at the area we were not observing. Besides, no further beam effect (e.g. amorphization, mass loss, and holing) was found during the growth of the nanoparticles. Importantly, the final product of the *in situ* STEM experiment, the octahedral Pt_3Ni nanoparticles show the same morphology and structure as the ex situ synthesized ones, indicating the shape development is not affected by the electron beam.

Supplementary Note 2: *In situ* STEM observation results discussion

As discussed above, to minimize the electron beam effect, we applied the low-dose STEM technique for the particle imaging, which sacrificed the image quality. This issue becomes more significant for the early stage small clusters considering the structural instability induced by the high mobility of the atoms at this stage. Previous studies have discovered that particles at cluster size are not thermodynamically stable in structure and could undergo transition between their polymorphic structures at high frequency [8-11]. This structural instability makes it difficult for clear imaging of the clusters. As can be seen in Fig. 1(a), the 1 image shows a quite blurred small cluster shadow. In actual imaging condition, it is highly dynamic and the captured images only shows a transient state. The instability of clusters and frequent transition between their polymorphic structures imply that they would likely be not able to maintain stable facets. Thus we think we cannot conclude there are facets for such unstable and dynamic clusters. In this regard, the model shown in Fig. 1 for early stage clusters is constructed from the STEM projection image and reflects only a captured transient state of the growing particle to better illustrate the facet evolution. To avoid possible misunderstandings, we added one statement in Fig. 1 caption: "The early stage model 1" is only constructed to show a possible dynamical cluster structure." And this will not affect our conclusions.

For the continuous imaging of the growing particles, the technological limitations and difficulty of *in situ* STEM make it very challenging to image multiple particles at a

same time or in a same experiment. This is because when we focus on one specific nanoparticle and continuously capture the details of its growing process, other nanoparticles would be out of observation range owing to a very small field of view at high magnification. This issue becomes even more challenging when we consider zone-axis orientation of the particles of observation, instability and mobility of the particles during the reaction, etc. On the other side, lattice and morphological details of the growing particles (especially at their early growth stage) would be sacrificed when multiple particles are imaged at a same time at a lower magnification. So in order to make sure the growth trend shown in Fig. 1 is reliable and generic, we did repeat our *in situ* STEM experiments for multiple times and imaged the growth details of multiple nanoparticles. We have shown the growth process of one different particle in Supplementary Fig. 4, 5 where the facet growth trend is consistent with the result from Fig. 1, suggesting the result in Fig. 1 is representative.

Besides imaging the growth details of individual particles, we have also tried to image multiple growing particles at a same time. Although we were not able to get the lattice information and the morphology information at the early stage due to a low magnification for imaging, we obtained a generic trend of the particle growth rate. The average size of the growing nanoparticles shows a combined growth trend of (001) and (111) facets and is comparable with the trend in Fig. 1 and Supplementary Fig. 4, 5. The growing particles at any given designated reaction time seems fairly uniform, evidenced by the small size deviations (shown in Supplementary Fig. 6(b)). And we also show detailed TEM images and size distribution of the particles at different growth stage to prove the homogenous distribution of the nanoparticles. As we can see in Supplementary Fig. 7, the particles are relatively homogenous in size. And the shapes are similar with each other. Also we have to note that when the particles grow larger, they become more faceted which leads to a bigger distribution broadening due to different zone-axis orientation of the shaped particles. Specifically, as to the octahedra yield, since for the growing nanoparticles, it's hard to specify if it's growing into octahedral at low magnification, so a late stage shape counting is more reasonable. As shown in Supplementary Fig. 8, we counted the identifiable octahedral particles for the late stage growth. About 77% of the grown particles possess an octahedral morphology, suggesting a high yield of shaped Pt_3Ni. This is consistent with our previous studies on this synthesis technique [12]. Thus, based on all these *in situ* STEM results, we think the discussions and conclusions we drew from Fig. 1 is representative and reliable.

<u>Supplementary Note 3: AP-XPS intensity and sampling depth discussion</u>

The intensity of the collected AP-XPS spectra shows a temperature-dependent correlation where the overall intensity of the spectra changes with increasing temperature. This phenomenon is related to the temperature of the specimen and the gas conditions used for the *in situ* AP-XPS study, which can be seen in previous studies [13, 14]. However, this correlation is beyond the scope of this work, and based on previous studies, the variations in overall intensity show negligible effect on the peak positions in this case. More importantly, the Pt 4*f* and Ni 2*p* spectra were continuously recorded at the same specific temperature, thus the Pt/Ni composition ratio at a particular temperature is not affected by these variations in the absolute intensity.

The AP-XPS spectra were collected at the photon energy of 1100 eV for both Pt 4*f* and Ni 2*p*. Thus based on the kinetic energies of the photoelectrons that are around 1028 eV for Pt 4*f* and 245 eV for Ni 2*p*, the inelastic mean free path (IMFP) are approximate 13.3 Å for metallic Pt and 6.1 Å for metallic Ni [15]. In this way, the Pt signal is more bulk sensitive while the Ni signal is more surface sensitive in our experiment, which means that we overestimate the Pt^0 composition ratio showed in Fig. 2(d). So the exact Ni^0/Pt^0 ratio will be higher than the values calculated from our experiment, which indicates a more severe surface Ni segregation. This is still consistent with our current conclusion though the accurate composition is not available. In a word, the data shown in Fig. 2 are adequate and acceptable to reveal the surface Ni segregation result.

<u>Supplementary Note 4: Discussion on temperature profile differences between experiments</u>

Three different *in situ* experiments (i.e. *in situ* STEM, AP-XPS and *in situ* XAS) are conducted with different temperature profiles in this paper due to their current technique limitations. For the AP-XPS experiments, the technique allowed us to use the temperature profile that was close to the one being used for solid-state synthesis of octahedral Pt_3Ni nanoparticles in our previous studies. However for the *in situ* STEM experiments, a same temperature profile would cause problems for imaging. This is because there are always thermo-induced expansions and drifts associated with temperature change, which would not allow imaging of the specimen with high resolution and quality. Thus the experiment had to be compromised at one steady temperature in order to image the growth process of individual particles. For the *in situ* XAS experiments, the beamline setup requires approximately 15 minutes to collect one XAS spectrum, which is too slow to monitor the particle growth process at a higher temperature at which the reduction occurs rapidly. In another word, we would not be able to collect sufficient time-series spectra for study. Thus in our experiment, we adopted a lower reaction temperature to slow down the growth kinetics. Although these technological limitations forced us to implement different temperature profiles, we found these discrepancies did not influence the growth mechanism of octahedral Pt_3Ni and thus did not invalidate the combinational uses of these technics to understand the growth mechanism. This conclusion was supported by the conducted *in situ* XAS experiments following both types of profiles (i.e. varying temperature profile and fixed temperature profile). The data were shown in Supplementary Fig. 14 and 15, which exhibited similar results that reveals a same particle growth mechanism. Moreover, octahedral Pt_3Ni nanoparticles were obtained from these experiments regardless of the different temperature profiles (Supplementary Fig. 3, 9). Thus it seems reasonable and acceptable to combine the information achieved from *in situ* STEM, AP-XPS and *in situ* XAS experiments to help understand the growth mechanism of octahedral Pt_3Ni.

<u>Supplementary Note 5: Reaction gas condition difference discussion</u>

Three different *in situ* experiments are conducted in this paper and due to the current technique limitations, the testing conditions are varied depending on the technology. In this case, the reaction gas conditions are different in STEM, AP-XPS and XAS experiments. To our knowledge, these differences are not crucial in the Pt_3Ni growth mechanism and we can get the targeted octahedral shape nanoparticles for all of these

experiments as confirmed in Supplementary Fig. 3, 9(a) and 9(b). To further validate this conclusion, we also conducted one more experiment with a very lower H_2 and CO partial pressure (0.5% H_2, 2.5% CO and 97% Ar, ultra-high purity, 1 atm) and we can still get desired octahedral Pt_3Ni nanoparticles shown in Supplementary Fig. 9(c). Thus, the gas condition differences are acceptable, and by combining these techniques the octahedral Pt_3Ni growth pathway can be revealed.

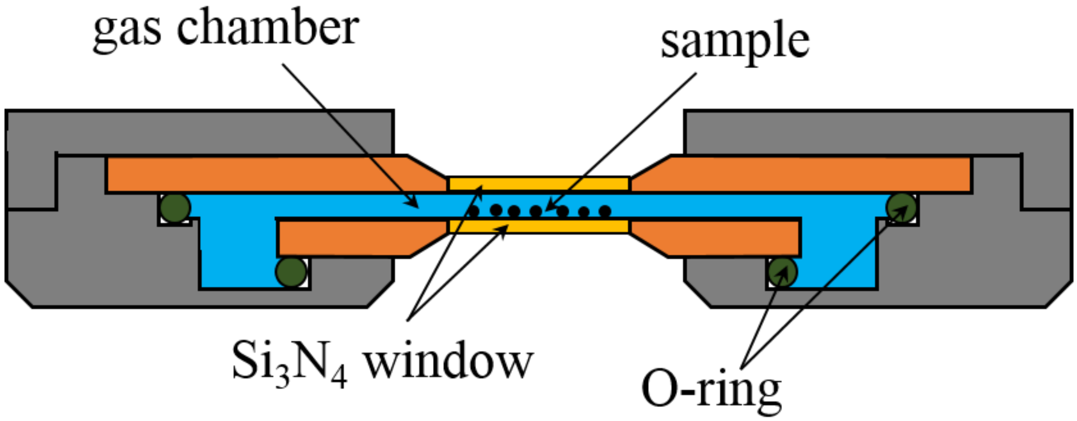

Supplementary Figure 1. *In situ* STEM gas cell structure.

Scheme of the cross section view of the *in situ* STEM gas cell based on the previous report. [16]

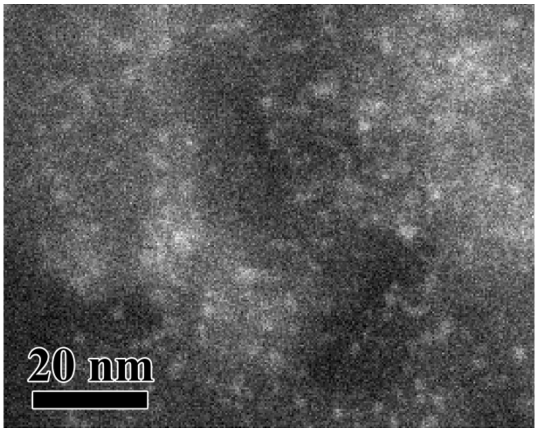

Supplementary Figure 2. Pt_3Ni nanoparticle nucleation.
Nucleation of Pt_3Ni nanoparticles under the electron beam illumination.

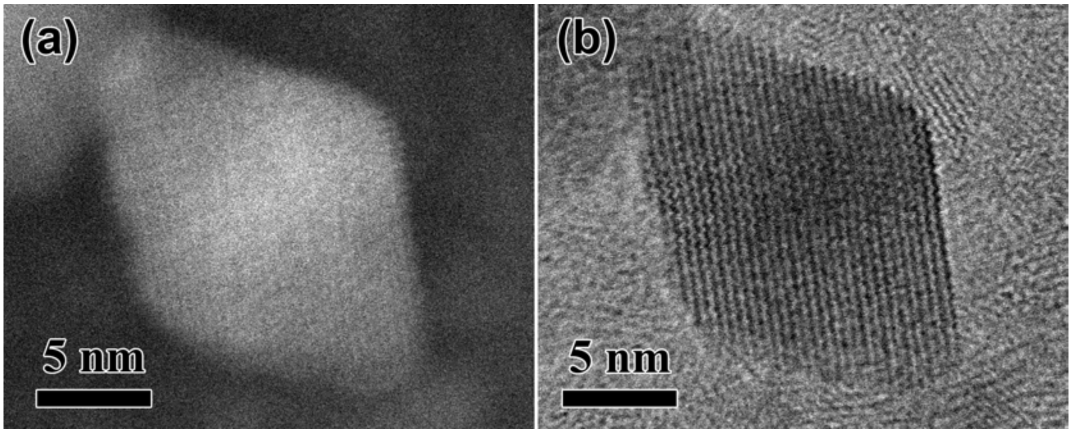

Supplementary Figure 3. Typical Pt_3Ni octahedron.

One typical Pt_3Ni octahedral nanoparticle at final stage: (a) dark field and (b) bright field STEM images.

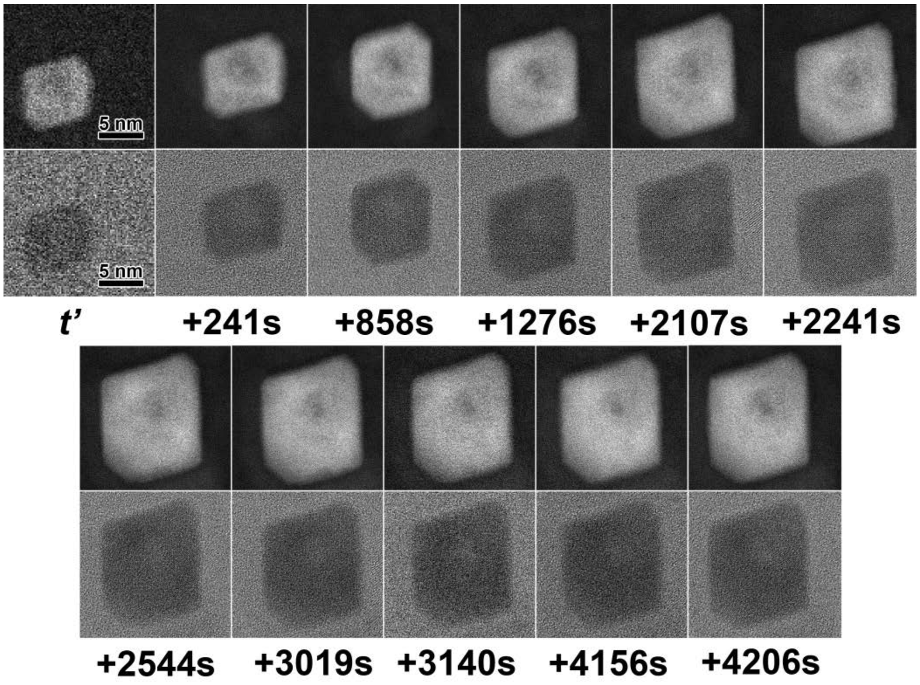

Supplementary Figure 4. Morphology evolution of one Pt_3Ni octahedron.

Sequential observation images of one Pt_3Ni octahedral nanoparticle growth: Dark field image (upper) and corresponding bright field image (lower).

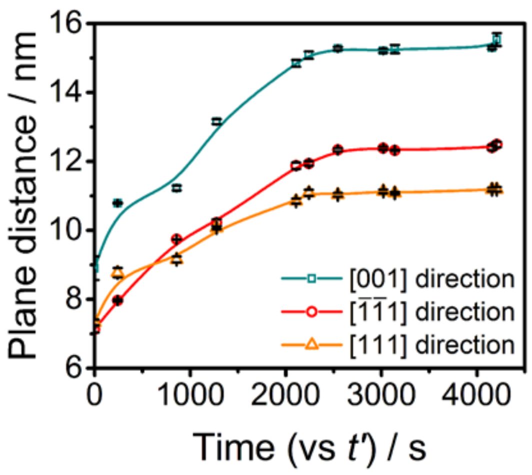

Supplementary Figure 5. Plane distance evolution plots of Pt_3Ni octahedron.

Grown size plots of Pt_3Ni octahedral nanoparticle: Data points with error bars are measured from Supplementary Fig. 4. Error bars correspond to standard deviations of at least three independent length measurements.

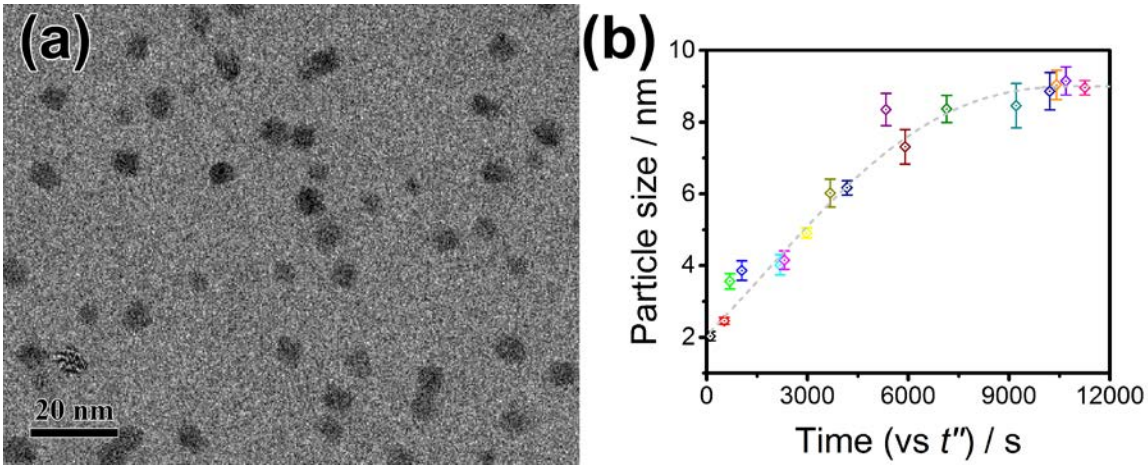

Supplementary Figure 6. Size distribution evolution during Pt_3Ni growth.

(a) Representative low-magnification STEM image of growing Pt_3Ni nanoparticles and (b) average size of the growing nanoparticles with reaction time (The time start of imaging is denoted as t’’). Error bars correspond to standard deviations of particle size distribution.

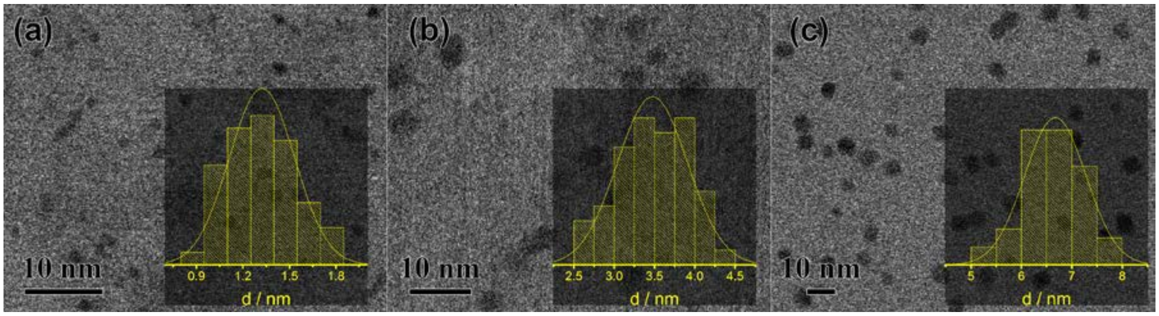

Supplementary Figure 7. Size distribution of Pt_3Ni at different growing stages. Typical low magnification STEM images and corresponding size distribution of the growing Pt_3Ni nanoparticles, (a), (b) and (c) corresponds to the early, middle and late stage of Pt_3Ni octahedra growth.

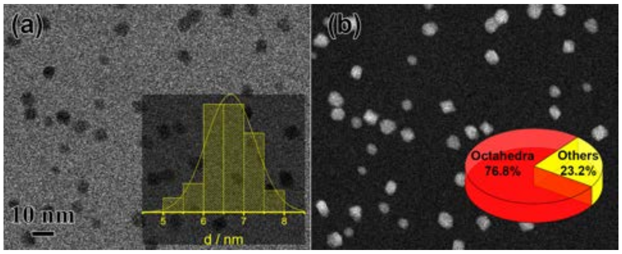

Supplementary Figure 8. Size distribution and octahedron yield of Pt_3Ni.

(a) Size distribution of Pt_3Ni at late stage and (b) corresponding estimated octahedra percentage. (Note: the particles counted in “others” section can also be octahedral but cannot be clearly identified due to the image quality and particle zone-axis orientation.)

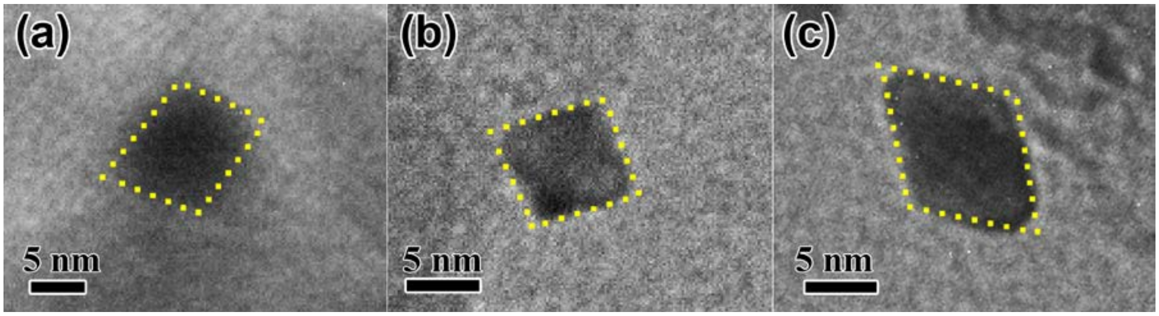

Supplementary Figure 9. Grown Pt_3Ni octahedron under various conditions.

Pt_3Ni octahedral nanoparticles from (a) APXPS experiment, (b) XAS experiment and (c) 0.5% H_2, 2.5% CO and 97% Ar gas condition experiment. Note that due to the rotation of the nanoparticle, the projection image could be slightly different for an octahedral nanoparticle.

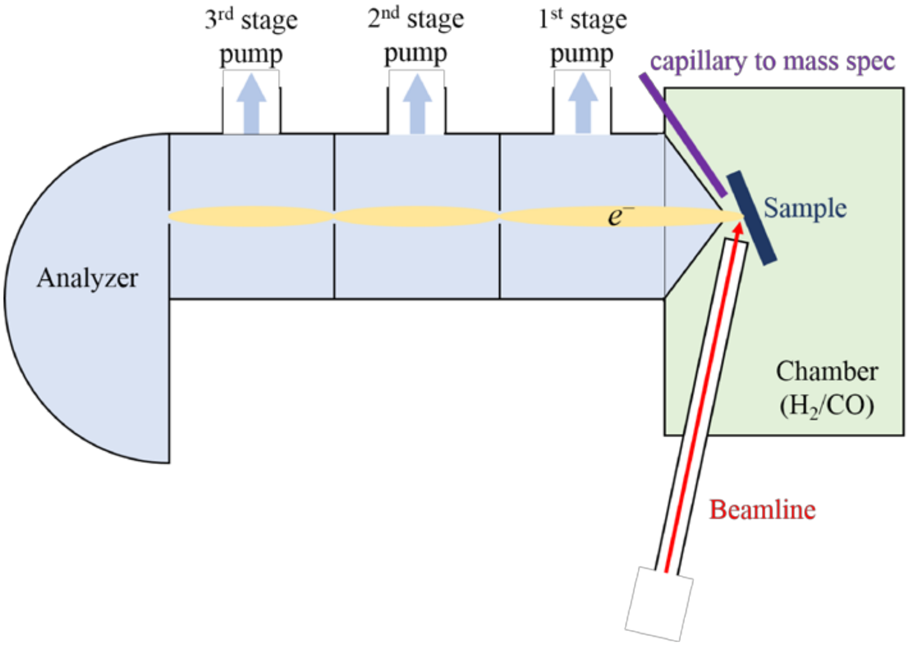

Supplementary Figure 10. *In situ* AP-XPS setup.
Scheme of the *in situ* AP-XPS setup based on the previous report. [17]

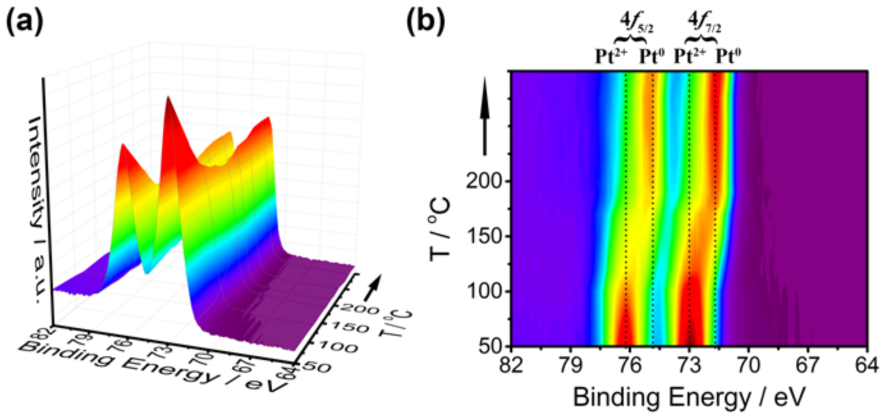

Supplementary Figure 11. AP-XPS spectra of pure Pt sample.

Temperature-dependent (a) Pt 4*f* spectra and (b) corresponding 2D projection spectra for pure Pt sample.

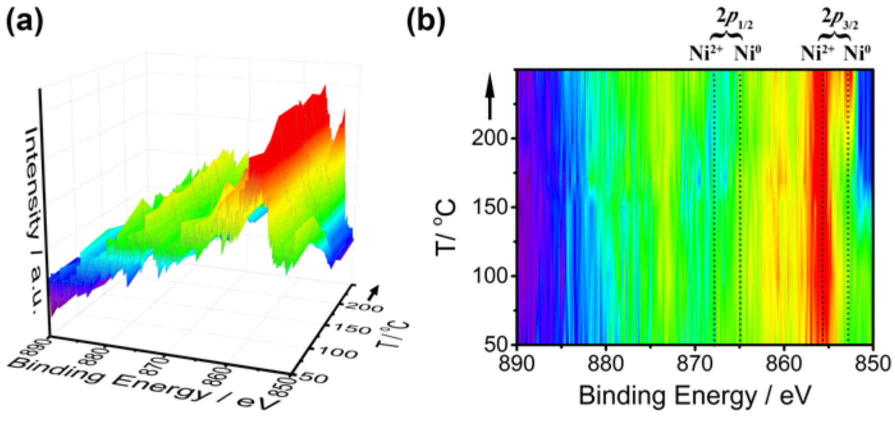

Supplementary Figure 12. AP-XPS spectra of pure Ni sample.

Temperature-dependent (a) Ni 2*p* spectra and (b) corresponding 2D projection spectra for pure Ni sample.

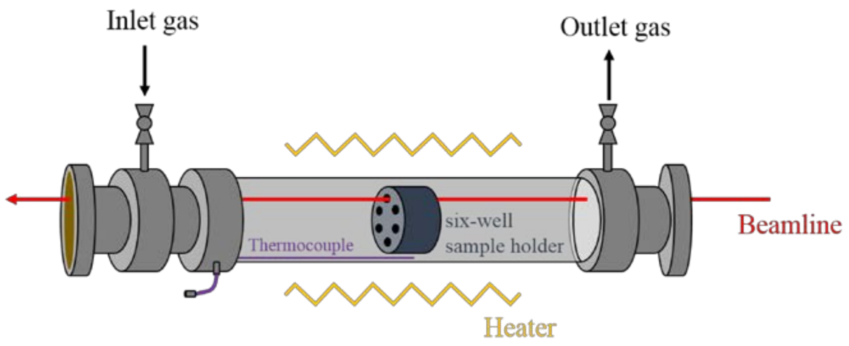

Supplementary Figure 13. ***In situ*** **XAS gas reactor structure.**
Scheme of the *in situ* XAS gas reactor based on the previous report. [18]

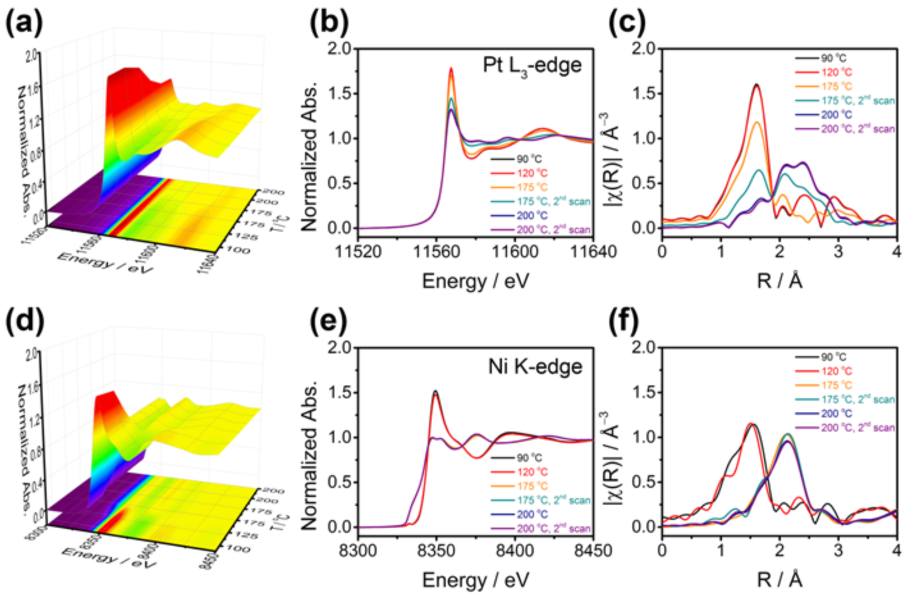

Supplementary Figure 14. Temperature-dependent XAS results of Pt_3Ni sample. *In situ* Pt L_3-edge spectra (a) and (b) with corresponding radial distance plot (c) at different temperatures for Pt_3Ni sample. *In situ* Ni K-edge spectra (d) and (e) with corresponding radial distance plot (f) at different temperatures for Pt_3Ni sample.

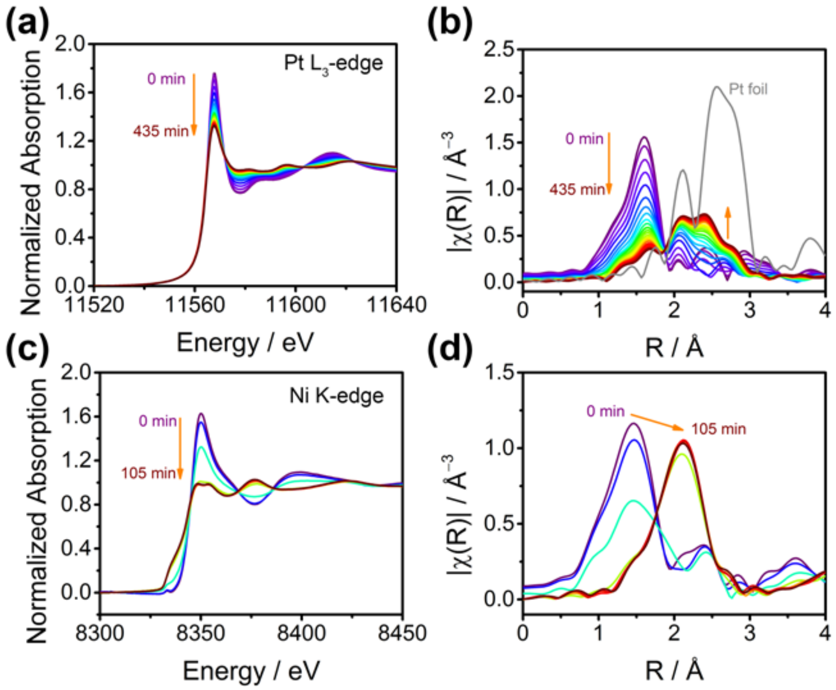

Supplementary Figure 15. XAS results of Pt_3Ni sample at 160 ^oC.

In situ Pt L_3-edge spectra (a) with corresponding radial distance plot (b) at 160 ^oC of Pt_3Ni sample. *In situ* Ni K-edge spectra (c) with corresponding radial distance plot (d) at 160 ^oC of Pt_3Ni sample.

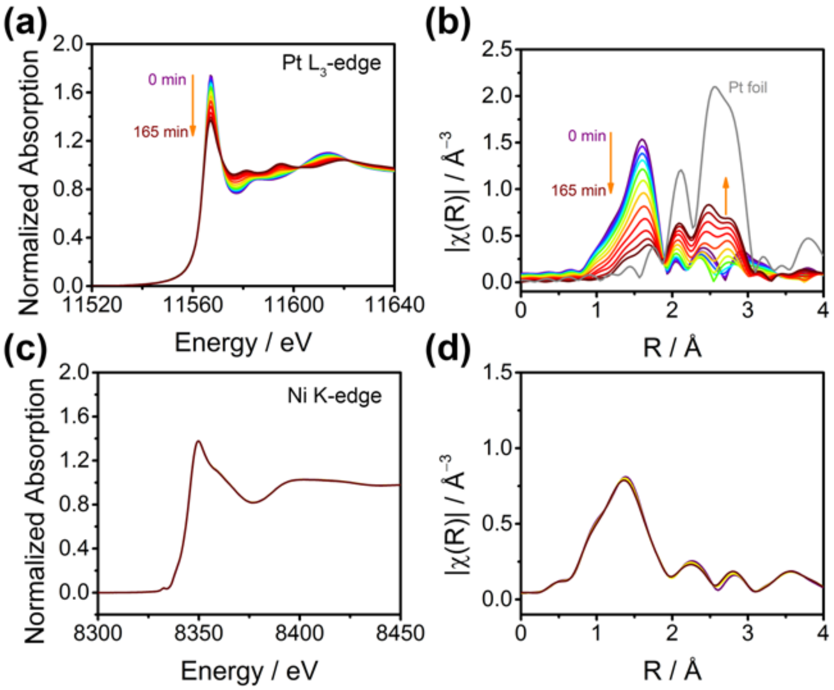

Supplementary Figure 16. XAS results of pure Pt and Ni samples at 160 ^{o}C.

In situ Pt L_3-edge spectra (a) with corresponding radial distance plot (b) at 160 ^{o}C of pure Pt sample. *In situ* Ni K-edge spectra (c) with corresponding radial distance plot (d) at 160 ^{o}C of pure Ni sample.

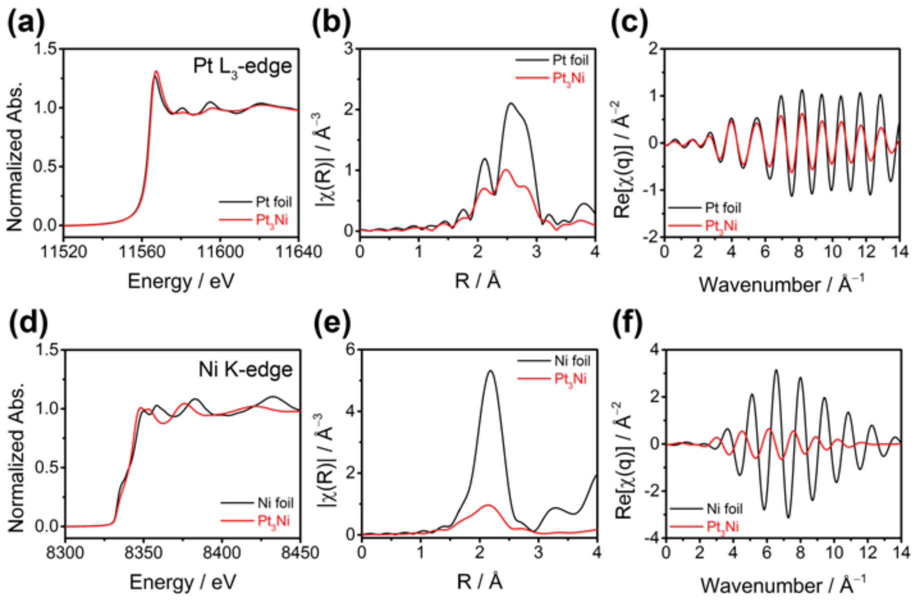

Supplementary Figure 17. Comparison of XAS results of Pt_3Ni sample with foil. Pt L_3-edge spectra (a) with corresponding radial distance plot (b) and Fourier-filtered q space plot (c) for comparison between Pt foil and Pt_3Ni sample. Ni K-edge spectra (d) with corresponding radial distance plot (e) and Fourier-filtered q space plot (f) for comparison between Ni foil and Pt_3Ni sample.

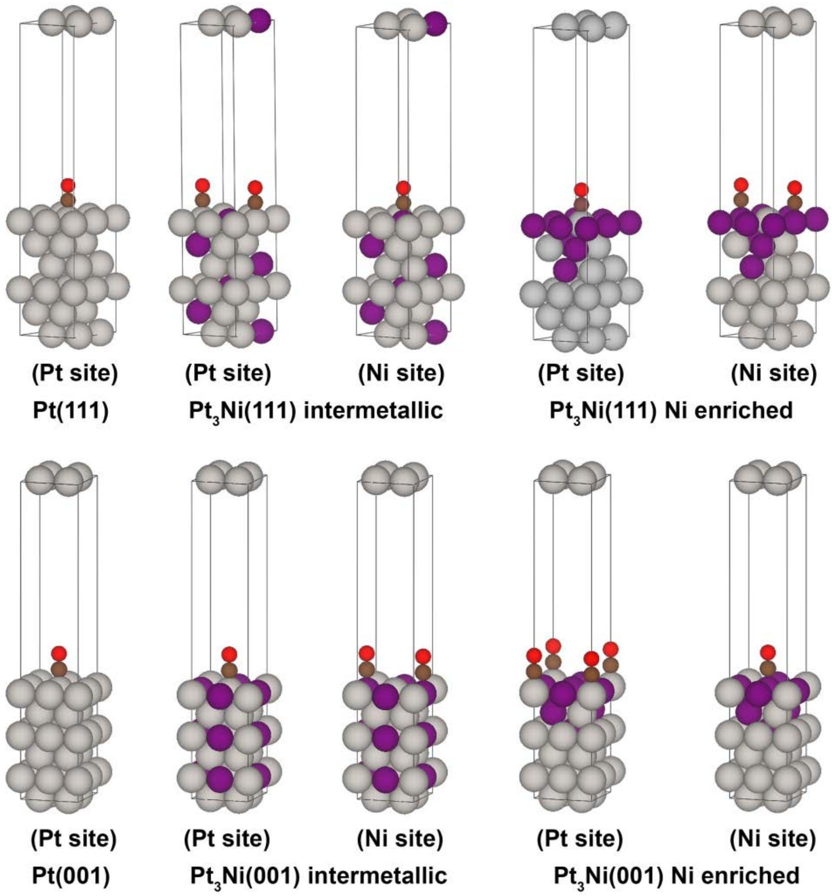

Supplementary Figure 18. DFT simulation models.

DFT simulation models used in this study. Note that the CO molecule at the side or corner is counted as 1/2 or 1/4 of molecule, the total number of CO belonging to the supercell is 1. (gray: Pt; purple: Ni; brown: C; red: O)

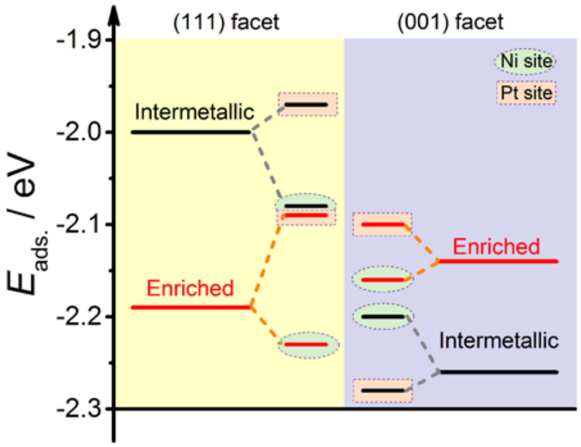

Supplementary Figure 19. CO adsorption energy comparison.

Comparison of CO adsorption energy on (111) and (001) facets of intermetallic Pt_3Ni and surface Ni enriched Pt_3Ni. The longer bar indicates the weighted adsorption energy ($E_{ads,w}$) calculated from the adsorption energy from corresponding Pt and Ni site adsorption (short bar).

Supplementary Table 1.

EXAFS fitting results for Pt and Ni foil reference, and Pt L_3-edge and Ni K-edge of final Pt_3Ni octahedral sample.

		S_0^2	C.N.	Distance / Å	ΔE_0	σ^2
Ni foil	Ni-Ni	0.787±0.017	12(fixed)	2.48±0.01	7.0±0.2	0.006±0.001
Pt foil	Pt-Pt	0.829±0.023	12(fixed)	2.76±0.01	7.8±2.7	0.005±0.001
Ni edge: Δk=3-11 $Å^{-1}$, ΔR=1.20-2.70 Å, R-factor: k^1=0.0003; k^2=0.0002; k^3=0.0002<br>Pt edge: Δk=3-14 $Å^{-1}$, ΔR=1.57-3.11 Å, R-factor: k^1=0.0015; k^2=0.0010; k^3=0.0012						
Pt edge (Pt_3Ni)	Pt-Pt	0.829	6.5±0.7	2.72±0.01	2.7±0.6	0.010±0.001
	Pt-Ni		3.1±0.9	2.61±0.01	2.7±0.6	0.018±0.003
Ni edge (Pt_3Ni)	Ni-Ni	0.787	3.4±1.7	2.56±0.02	-8.0±2.7	0.011±0.004
	Ni-Pt		7.1±3.9	2.61±0.01	-8.0±2.7	0.018±0.003
Ni edge: Δk=3-11 $Å^{-1}$, ΔR=1.42-3.00 Å, R-factor: k^1=0.015; k^2=0.009; k^3=0.007<br>Pt edge: Δk=3-14 $Å^{-1}$, ΔR=1.80-3.30 Å, R-factor: k^1=0.004; k^2=0.004; k^3=0.007						

Supplementary Table 2.

Summary of the DFT simulated results.

**CO E_{ads} on (111) surface**			
**No. of Ni atoms***	**Pt**	**Pt_3Ni intermetallic**	**Pt_3Ni Ni enriched**
Layer 1	0	1	3
Layer 2	0	1	2
Layer 3	0	1	1
Layer 4	0	1	0
Layer 5	0	1	0
Layer 6	0	1	0
θ_{Pt}	1	0.75	0.25
θ_{Ni}	0	0.25	0.75
$E_{ads,Pt}$ / eV	-2.04	-1.97	-2.09
$E_{ads,Ni}$ / eV	--	-2.08	-2.23
$E_{ads,w}$ / eV	-2.04	-2.00	-2.19
**CO E_{ads} on (001) surface**			
**No. of Ni atoms***	**Pt**	**Pt_3Ni intermetallic**	**Pt_3Ni Ni enriched**
Layer 1	0	2	3
Layer 2	0	0	2
Layer 3	0	2	1
Layer 4	0	0	0
Layer 5	0	2	0
Layer 6	0	0	0
θ_{Pt}	1	0.75	0.25
θ_{Ni}	0	0.25	0.75
$E_{ads,Pt}$ / eV	-2.20	-2.28	-2.10
$E_{ads,Ni}$ / eV	--	-2.20	-2.16
$E_{ads,w}$ / eV	-2.20	-2.26	-2.14

*Layers are labeled from top surface to bottom.

Supplementary References

1. Batson, P. E. Motion of gold atoms on carbon in the aberration-corrected STEM. *Microsc. Microanal.* **14**, 89-97 (2007).
2. Krivanek, O. L., *et al.* Gentle STEM: ADF imaging and EELS at low primary energies. *Ultramicroscopy* **110**, 935-945 (2010).
3. Aindow, M., Kiely, C. J. *Electron Microscopy and Analysis 2001*. CRC Press (2001).
4. Buban, J. P., Ramasse, Q., Gipson, B., Browning, N. D., Stahlberg, H. High-resolution low-dose scanning transmission electron microscopy. *J. Electron. Microsc.* **59**, 103-112 (2010).
5. Zhang, S., *et al.* Revealing particle growth mechanisms by combining high-surface-area catalysts made with monodisperse particles and electron microscopy conducted at atmospheric pressure. *J. Catal.* **337**, 240-247 (2016).
6. Su, D. S., *et al.* Electron beam induced reduction of V_2O_5 studied by analytical electron microscopy. *Catal. Lett.* **75**, 81-86 (2001).
7. Liao, H.-G., *et al.* Facet development during platinum nanocube growth. *Science* **345**, 916-919 (2014).
8. Dirk, Z. Thermodynamics and kinetics of prenucleation clusters, classical and non-classical nucleation. *ChemPhysChem* **16**, 2069-2075 (2015).
9. Chen, F., Curley, B. C., Rossi, G., Johnston, R. L. Structure, melting, and thermal stability of 55 atom Ag−Au nanoalloys. *J. Phys. Chem. C* **111**, 9157-9165 (2007).
10. Wang, J., Wang, G., Zhao, J. Density-functional study of Au_n(n=2--20) clusters: Lowest-energy structures and electronic properties. *Phys. Rev. B* **66**, 035418 (2002).
11. Gebauer, D., Cölfen, H. Prenucleation clusters and non-classical nucleation. *Nano Today* **6**, 564-584 (2011).
12. Zhang, C., Sandorf, W., Peng, Z. Octahedral Pt_2CuNi uniform alloy nanoparticle catalyst with high activity and promising stability for oxygen reduction reaction. *ACS Catal.* **5**, 2296-2300 (2015).
13. Cui, C., *et al.* Shape-selected bimetallic nanoparticle electrocatalysts: evolution of their atomic-scale structure, chemical composition, and electrochemical reactivity under various chemical environments. *Faraday Discuss.* **162**, 91-112 (2013).
14. Zhao, Y., *et al.* Oxide-modified nickel photocatalysts for the production of hydrocarbons in visible light. *Angew. Chem. Int. Ed.* **55**, 4215-4219 (2016).
15. Tanuma, S., Powell, C. J., Penn, D. R. Calculations of electorn inelastic mean free paths. II. Data for 27 elements over the 50–2000 eV range. *Surf. Interface Anal.* **17**, 911-926 (1991).
16. Dai, S., Gao, W., Zhang, S., Graham, G. W., Pan, X. Transmission electron microscopy with atomic resolution under atmospheric pressures. *MRS Commun.* **7**, 798-812 (2017).
17. Palomino, R. M., *et al.* New in-situ and operando facilities for catalysis science at NSLS-II: The deployment of real-time, chemical, and structure-sensitive X-ray probes. *Synchrotron Radiat. News* **30**, 30-37 (2017).
18. Setthapun, W., *et al.* Genesis and evolution of surface species during Pt atomic layer deposition on oxide supports characterized by in situ XAFS analysis and water−gas shift reaction. *J. Phys. Chem. C* **114**, 9758-9771 (2010).
